# SOD1 aggregation in astrocytes following ischemia/reperfusion injury: a role of NO-mediated S-nitrosylation of protein disulfide isomerase (PDI)

**DOI:** 10.1186/1742-2094-9-237

**Published:** 2012-10-12

**Authors:** Xueping Chen, Teng Guan, Chen Li, Huifang Shang, Liying Cui, Xin-Min Li, Jiming Kong

**Affiliations:** 1 Department of Human Anatomy and Cell Science, University of Manitoba, 745 Bannatyne Avenue, Winnipeg, Manitoba, R3E 0J9, Canada; 2 Department of Neurology, West China Hospital, Sichuan University, Chengdu, 610041, China; 3 Department of Neurology, Beijing Union Hospital, Beijing, China; 4 Department of Psychiatry, Faculty of Medicine, University of Manitoba, Winnipeg, Manitoba, Canada

**Keywords:** Nitrosative stress, PDI, S-nitrosylation, SOD1, Ubiquitinated-protein aggregates

## Abstract

**Background:**

Ubiquitinated-protein aggregates are implicated in cerebral ischemia/reperfusion injury. The very presence of these ubiquitinated-protein aggregates is abnormal and seems to be disease-related. However, it is not clear what leads to aggregate formation and whether the aggregations represent a reaction to aggregate-mediated neurodegeneration.

**Methods:**

To study the nitrosative stress-induced protein aggregation in cerebral ischemia/reperfusion injury, we used primary astrocyte cultures as a cell model, and systematically examined their iNOS expression and consequent NO generation following oxygen glucose deprivation and reperfusion. The expression of protein disulfide isomerase (PDI) and copper-zinc superoxide dismutase (SOD1) were also examined, and the biochemical interaction between PDI and SOD1 was determined by immunoprecipitation. In addition, the levels of S-nitrosylated PDI in cultured astrocytes after oxygen glucose deprivation and reperfusion treatment were measured using the biotin-switch assay. The formation of ubiquitinated-protein aggregates was detected by immunoblot and immunofluorescence staining.

**Results:**

Our data showed that the up-regulation of iNOS expression after oxygen glucose deprivation and reperfusion treatment led to excessive NO generation. Up-regulation of PDI and SOD1 was also identified in cultured astrocytes following oxygen glucose deprivation and reperfusion, and these two proteins were found to bind to each other. Furthermore, the increased nitrosative stress due to ischemia/reperfusion injury was highly associated with NO-induced S-nitrosylation of PDI, and this S-nitrosylation of PDI was correlated with the formation of ubiquitinated-protein aggregates; the levels of S-nitrosylated PDI increased in parallel with the formation of aggregates. When NO generation was pharmacologically inhibited by iNOS specific inhibitor 1400W, S-nitrosylation of PDI was significantly blocked. In addition, the formation of ubiquitinated-protein aggregates in cultured astrocytes following oxygen glucose deprivation and reperfusion was also suppressed by 1400W. Interestingly, these aggregates were colocalized with SOD1, which was found to co-immunoprecipitate with PDI.

**Conclusions:**

NO-mediated S-nitrosylation of PDI may be involved in the formation of the SOD1-linked ubiquitinated-protein aggregates in cerebral ischemia/reperfusion injury.

## Background

Brain ischemia/reperfusion injury is a major public health problem. It causes excitotoxicity, inflammation, cell death, and compensatory neurogenesis [1,2]. Neurons are more susceptible to hypoxic stress than astrocytes. They have fewer antioxidant mechanisms than astrocytes and rely mainly on the metabolic support from surrounding astrocytes [2-4]. It is proposed that brain ischemia/reperfusion injury is a consequence of the failure of astrocytes to support the essential needs of neurons. Dysfunction of astrocytes may lead to increasing neuronal death [2]. Any pathophysiological events that affect the function of astrocytes would therefore compromise their neuronal supportive role. One detrimental event after ischemia/reperfusion injury is the dramatic increase in damaging free radicals, such as nitrogen oxide (NO), superoxide, and peroxynitrite [5]. Expressions and activities of nitrogen oxide synthases (NOS) are enhanced in the experimental mouse model of cerebral ischemia/reperfusion injury. Nitrogen oxide and its further oxidative products are generally implicated in the pathology of brain ischemia/reperfusion injury. There are three isoforms of mammalian NOS: neuronal NOS (nNOS), inducible NOS (iNOS), and endothelial NOS (eNOS). Although experimental brain ischemia/reperfusion injury leads to the up-regulation of all three NOS isoforms, their expression patterns differ both temporally and spatially. The induction of iNOS expression occurs much later than nNOS and eNOS, suggesting that iNOS contributes to relatively late injury [6]. iNOS-deficient mice exhibit a significant reduction in infarct volume and attendant behavioral change after 48 h of hypoxic or ischemic injury [7,8]. Studies show that astrocytes are the cells mainly responsible for iNOS expression after ischemia/reperfusion injury [9]. Once iNOS is expressed following transient hypoxia or ischemia, it will promote the production of neurotoxic amounts of NO [10,11], with maximal levels after 24 h in the striatum and 48 h in the cortex, without any requirement for further activation [12].

Ischemia/reperfusion injury may impair chaperone function and ubiquitin-proteasomal degradation and lead to protein aggregation [13]. Studies show that ubiquitinated-protein aggregates can be visualized in cultured astrocytes after glucose deprivation [14]. Ischemia/reperfusion injury disrupts proper peptide folding in the endoplasmic reticulum (ER) and triggers ER stress and an unfolded protein response [15]. The formation of these ubiquitinated-protein aggregates is one of the consequences of functional disturbance within the ER. The proper maturation and folding of native proteins rely on the activity of the ER chaperones and enzymes. Dysfunction of ER chaperone proteins can lead to protein misfolding and further aggregation, which will be recognized and ubiquitinated by the ubiquitin system through a series of ATP-dependent reactions [16]. Protein disulfide isomerase (PDI), an ER chaperone, is critical for proper protein folding in the ER. Protein disulfide isomerase is responsible for facilitating disulfide bond formation, rearrangement reactions, and structural stability [17]. Copper-zinc superoxide dismutase (SOD1) is an intracellular homodimeric metalloprotein, which is stabilized by an intrasubunit disulfide bond between cysteine 57 and cysteine 146. Mutations in SOD1 protein promote the formation of disulfide-reduced monomers, which are prone to forming aggregates. Hence, modulation of disulfide bond formation may be important in SOD1-linked aggregate formation. Protein disulfide isomerase was found to be associated with SOD1 in cellular and animal models of familial amyotrophic lateral sclerosis, a neurodegenerative disease affecting motor neurons. Furthermore, a biochemical interaction between PDI and SOD1 is implicated in the pathogenesis of familial amyotrophic lateral sclerosis [18]. In many neurodegenerative disorders and cerebral ischemia, up-regulation of PDI expression represents an adaptive response that promotes protein refolding and may offer neuronal cell protection [19,20]. Recently, Uehara and colleagues [21] demonstrated that in Parkinson’s disease and related neurodegenerative disorders, the NO-mediated S-nitrosylation of PDI inhibits PDI function, which leads to dysregulated protein folding, and consequently results in ER stress that promotes neuronal cell death. S-nitrosylation is an important biological reaction of NO and involves the covalent addition of NO to a cysteine thiol group of the protein to form S-nitrosothiols. This modification can affect many cellular processes and alter both protein function and protein-protein interactions [22].

In this study, we examined whether iNOS expression was correlated with NO-induced S-nitrosylation of PDI in cultured astrocytes following oxygen glucose deprivation (OGD)/reperfusion treatment. We also detected whether or not S-nitrosylation of PDI was associated with an accumulation of ubiquitinated-protein aggregates. We report here that the OGD/reperfusion treatment of cultured astrocytes led to an increase in NO production that was accompanied by augmented iNOS protein expression. The expression of both PDI and SOD1 were adaptively up-regulated in response to ischemia/reperfusion injury and an interaction between these two proteins was identified in cultured astrocytes by using co-immunoprecipitation. Although total PDI expression was increased following OGD/reperfusion treatment, PDI was found to be S-nitrosylated by ischemia/reperfusion-induced nitrosative stress. The formation of S-nitrosylated PDI (SNO-PDI) was detected in cultured astrocytes following OGD/reperfusion treatment, and the SNO-PDI level had a parallel relationship with the formation of ubiquitinated-protein aggregates. These aggregates were found to be colocalized with SOD1 protein, which was indicated to be a ubiquitinated protein in astrocytes under ischemia/reperfusion stress. Blocking NO generation with iNOS inhibitor 1400W significantly attenuated the formation of SNO-PDI and ubiquitinated-protein aggregates in cultured astrocytes following OGD/reperfusion treatment. We report here that, in cultured astrocytes, the up-regulation of iNOS after OGD/reperfusion promoted the NO-mediated S-nitrosylation of PDI. This modification of PDI may affect the chaperone activity of PDI and result in the formation of SOD1-linked ubiquitinated-protein aggregates in cultured astrocytes.

## Materials and methods

### Primary astrocyte cell culture

Primary astrocytes were taken from cerebral cortices of neonatal Wistar rats, as described previously [23]. Cortices were harvested, while the meninges and blood vessels were removed. Tissues were digested in 0.25% trypsin containing 0.1 M EDTA at 37°C for 15 min, and passed through a nylon sieve (80 μm pore size). The cells were seeded in Dulbecco’s modified Eagle’s medium supplemented with heat-inactivated 10% fetal calf serum, 50 μg/ml penicillin, and 100 μg/ml streptomycin. Cultured cells were grown at 37°C in a humidified atmosphere with 5% CO_2_. After 10 days, the microglia and oligodendrocyte progenitors were depleted by shaking. The remaining astrocytes were then detached by trypsinization and re-plated at a density of approximately 1 × 10^5^ cells/ml for future experiments. The purity of astrocytes (>90%) was identified by immunohistochemical analysis with anti-glial fibrillary acidic protein.

### OGD/reperfusion and 1400W treatment

On the third day of subculture, astrocytes were subjected to OGD with Earl’s balanced salt solution (EBSS) (6,800 mg/l NaCl, 400 mg/l KCl, 264 mg/l CaCl_2_·2H_2_O, 200 mg/l MgCl_2_·7H_2_O, 2,200 mg/l NaHCO_3_, 140 mg/l NaH_2_PO_4_·H_2_O, pH 7.2) and incubated in a hypoxic incubator filled with 1.5% O_2_ and 5% CO_2_ for 8 h. The oxygen level in the OGD solution decreased to about 2% to 3% after 60 min in the hypoxic incubator [24]. The cells were then provided with a normal amount of oxygen and maintenance medium without glutamate to mimic *in vivo* reperfusion for up to 24 h. Normoxic control cells were incubated in 37°C with 5% CO_2_ and atmospheric air in a buffer almost identical to EBSS except containing 5.5 mM glucose. iNOS inhibitor 1400W was prepared as a concentrated stock solution according to the manufacturer’s instructions. The final concentrations of 1400W in media applied to astrocytes were: 1, 10, and 50 μM. 1400W was added to culture medium 30 min prior to OGD exposure, and astrocytes were maintained in EBSS and maintenance medium during the treatment.

### Measurement of NO level

The concentration of NO in the culture medium was determined by the Griess reaction with minor changes [25]. Briefly, 40 μl cell culture fluid, 10 μl NADPH, and 40 μl basal solution (0.03 M PBS; 1.25 mM glucose-6-phosphate; 400 U/l glucose-6-phosphate dehydrogenase; 200 U/l nitrate reductase) were incubated in a 96-well microtiter plate for 45 min at room temperature. Next, 50 μl Griess reagent was added and the solution incubated for 20 min in the dark at room temperature. Finally, the absorbance of the samples was measured at 540 nm. NO_2_ concentrations were calculated from a standard curve of sodium nitrite (NaNO_2_).

### Western blot

Protein concentrations of cell lysates were determined by using the bicinchoninic acid (BCA) method (Pierce, Rockford). Samples (20 μg) were loaded on 12% sodium dodecyl sulphate polyacrylamide gel (SDS-PAGE) for electrophoresis and then transferred to the PVDF membrane. Membranes were blocked with 5% (w/v) milk in TBS-T buffer (10 mM Tris–HCl; pH 7.5; 150 mM NaCl; 0.05% Tween-20) for 1 h and then incubated with primary antibodies for 16 h at 4°C; SOD1 (1:2000, Santa Cruz Biotechnology), iNOS (1:1000, Santa Cruz Biotechnology), PDI (1:1000, Cedarlane). β-actin was used as an internal control (1:2000, Santa Cruz Biotechnology). Blots were washed in TBS-T buffer three times, probed with HRP-conjugated goat anti-rabbit or goat anti-mouse antibodies at 1:2500 for 1 h at room temperature, and then developed using chemiluminescence reagents (PerkinElmer). Quantification of band intensities was performed by densitometric analysis using Quantity One® 1-D analysis software (Bio-Rad).

### Immunoprecipitation

Whole-cell lysates (200 μl) were incubated with 50 μl of protein A-Sepharose CL-4B (Amersham Biosciences) for 30 min at 4°C with gentle rotation to remove IgG from the sample. The beads were briefly spun down and precleared cell lysates transferred to fresh tubes. 30 μl of 50% (w/v) protein A-Sepharose CL-4B in Tris buffer (50 mm Tris–HCl; pH 7.5;0.02% (w/v) NaN_3_) and anti-SOD1 or anti-PDI (1:750) antibodies were incubated with 100 μl precleared cell lysates on a rotating wheel overnight at 4°C. 20 μg of total protein was incubated with the sepharose-antibody to capture the antibody-binding protein complexes. After centrifugation at 15,800*g* for 1 min to remove the supernatant, the precipitate was washed three times in Tris buffer for 10 min each time. Both the supernatant and the immunoprecipitate was mixed with a 2% (w/v) SDS sample loading buffer and used for SDS-PAGE and immunoblot, following the methods described.

### Biotin-switch assay for detection of SNO-PDI

The cell lysates were prepared in HENC buffers (250 mM HEPES pH 7.5; 1 mM EDTA; 0.1 mM neocuproine; 0.4% CHAPS). Typically 1 mg of cell lysate was used. The blocking buffer (2.5% SDS, 20 mM methyl methane thiosulfonate (MMTS) in HEN buffer) was mixed with the samples and incubated for 30 min at 50°C to block any free thiol groups. After removing excess MMTS by acetone precipitation, nitrosothiols were reduced to thiols with 1 mM ascorbate. The newly formed thiols were then linked with the sulfhydryl-specific biotinylating reagent *N*-(6-(biotinamido)hexyl)-3^′^-(2^′^-pyridyldithio)propionamide (biotin-HPDP). The biotinylated proteins were pulled down with streptavidin-agarose beads. Western blot analysis was then performed to detect the amount of PDI remaining in the samples [26].

### Subcellular fractionation

Both the pellet and cytosolic fraction were prepared as described by Chen *et al*. [27]. Briefly, cell lysates of cultured astrocytes were sonicated for 30 s at 4°C in ice-cold lysis buffer (pH 7.6) containing: 15 mM Tris–HCl, 1 mM dithiothreitol, 250 mM sucrose, 1 mM MgCl_2_, 2.5 mM EDTA, 1 mM EGTA, 250 mM Na_3_VO_4_, 25 mM NaF, 2 mM sodium pyrophosphate, 0.5 mM phenylmethylsulfonyl fluoride, plus 1 μg/ml pepstatin A, 5 μg/ml leupeptin, and 2.5 μg/ml aproptonin. The protein content of the lysates was determined by BCA assay. Equal amounts of total cell lysate protein in each sample (0.5 mg) were centrifuged at 13,000*g* in 4°C for 10 min. The pellet fractions were sonicated three times and washed for 1 h at 4°C with 2% Triton X-100 and 150 mM KCl in the ice-cold lysis buffer. After being centrifuged at 13,000*g* in 4°C for 10 min, the pellet fraction containing the detergent and salt-insoluble aggregates was sonicated and redissolved in the lysis buffer for Western blotting.

### Double-immunofluorescence staining of ubiquitin and SOD1

Astrocytes on coverslips were fixed with 4% paraformaldehyde in PBS for 20 min. After being rinsed three times, cells were incubated with the blocking buffer (5% goat serum, 0.3% Triton X-100, and 1% bovine serum albumin in PBS) for 1 h, the coverslips were incubated with primary antibodies against ubiquitin (1:500) and SOD1 (1:1000) overnight at 4°C. After rinsing, coverslips were incubated with Alexa Fluor 488 anti-mouse IgG or 594 anti-rabbit IgG for 1 h at 37°C. Then Hoechst 33342 was added to stain the nuclei and the coverslips were mounted on the slides. The slides were imaged by an observer blind to treatments on a Zeiss Axiovert upright fluorescent microscope with identical exposure settings and identical post-acquisition processing for each image.

### Statistical analysis

Quantification of band intensities was performed by densitometric analysis using Quantity One® 1-D analysis software (Bio-Rad). The statistical calculations and graphing were performed using GraphPad Prism® software, version 5. All data were tested using one-way analysis of variance (ANOVA) with Tukey’s *post*-*hoc* test. A *P* value of 0.05 or less was judged significant. Results were expressed as mean ± SEM.

## Results

### OGD/reperfusion induces NO formation and iNOS protein expression

**Figure 1:**
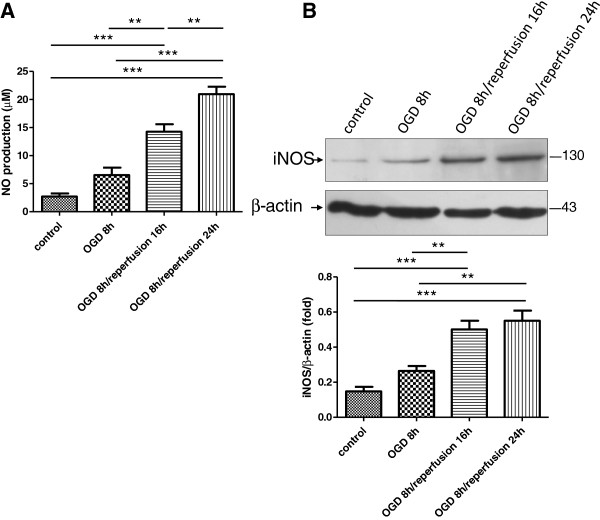
**Effect of OGD**/**reperfusion on NO production and iNOS expression.A**. The changes of NO levels in cultured astrocytes following OGD/reperfusion as determined by Griess reagent. Following reperfusion, NO production increased gradually and reached a peak under the treatment of OGD 8 h/reperfusion 24 h. This increase was significant in comparison with all other groups. **B**. iNOS protein levels in cultured astrocytes following OGD/reperfusion as determined by Western blotting. Full-length iNOS protein (~130 kDa) was more enriched in the OGD/reperfusion groups compared with the control group. OGD/reperfusion significantly induced greater levels of iNOS than in normal conditions. Five independent experiments were performed and data were analyzed in triplicate. Values were presented as mean ± SEM; *, *P* < 0.05; **, *P* < 0.01; ***, *P* < 0.001 by one-way ANOVA (analysis of variance) with Tukey’s *post*-*hoc* test.

The cortical astrocyte culture was subjected to OGD for 8 h and then exposed to reperfusion for either 16 h or 24 h. The concentration of NO in the culture media and the protein expression of iNOS were then determined. Nitrogen oxide generation was slightly increased after OGD treatment. The increase of NO was exaggerated by the reperfusion treatment. The concentration of NO reached a maximal level at the latest time studied (OGD 8 h/reperfusion 24 h) (Figure 1A). A similar result was observed in the iNOS protein assay. Under normal circumstances, the iNOS expression was too low to be detected. After OGD treatment, the iNOS expression was up-regulated. The reperfusion treatment led to a dramatic increase of iNOS expression in reactive astrocytes compared with the control cells that were not treated. When iNOS protein expression was quantified, exposure to OGD/reperfusion induced a remarkable increase in the iNOS protein level, especially in astrocytes following OGD 8 h/reperfusion 24 h. These cells exhibited a significant increase of iNOS expression, about 2.5-fold as compared with the control (Figure 1B).

### PDI and SOD1 are up-regulated after OGD/reperfusion treatment, and they were binding to each other

**Figure 2:**
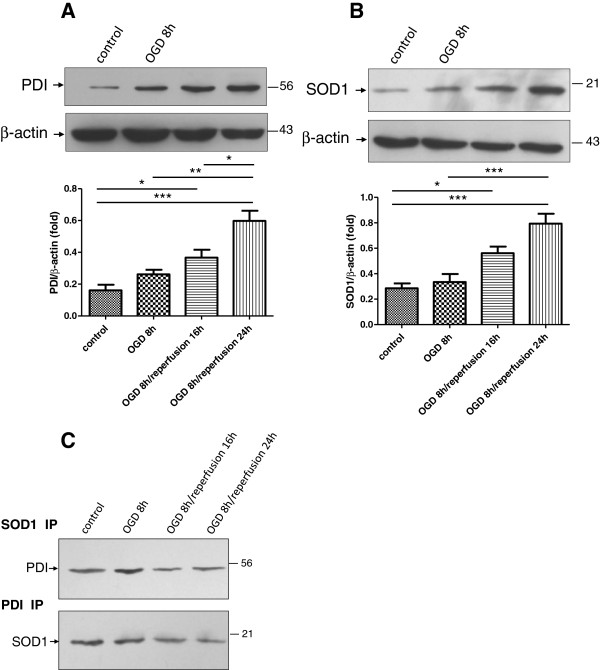
**Expression of PDI and SOD1 in cultured astrocytes following OGD**/**reperfusion.A**. PDI expression in cultured astrocytes showed an increasing trend in the process of reperfusion and peaked at OGD 8 h/reperfusion 24 h. **B**. SOD1 expression in cultured astrocytes increased gradually during the process of OGD/reperfusion and reached a peak at OGD 8 h/reperfusion 24 h. **C**. The anti-SOD1 antibody co-precipitated PDI, and the anti-PDI antibody co-precipitated SOD1 in cultured astrocytes. Three separate immunoprecipitations were performed, and data were analyzed in triplicate. Data were presented as mean ± SEM; *, *P* < 0.05; **, *P* < 0.01; ***, *P* < 0.001 by one-way ANOVA (analysis of variance) with Tukey’s *post*-*hoc* test.

We investigated the changes in PDI and SOD1 expression levels following OGD/reperfusion treatment in cultured astrocytes. Cell lysates from astrocytes under various treatments were analyzed by immunoblot. Western blot analysis using anti-PDI monoclonal antibody revealed an enhancement of PDI expression after OGD at 8 h, and this had reached a maximum by 24 h of reperfusion (Figure 2A). A similar pattern was also observed in SOD1 expression. Immunoblot analyses confirmed an increased expression of SOD1 in cultured astrocytes when they were exposed to OGD for 8 h. In addition, reperfusion significantly induced appreciable SOD1 expression in these cells, yielding a three-fold higher abundance of SOD1 protein in the OGD 8 h/reperfusion 24 h group when compared with the control group (Figure 2B). These results demonstrate that the elevation of PDI and SOD1 expression correlate well with the induction of iNOS in activated astrocytes following OGD/reperfusion treatment. We then used immunoprecipitation to examine the interaction between PDI and SOD1. Cell lysates from astrocytes under various treatments were subjected to immunoprecipitation using the anti-SOD1 antibody or anti-PDI antibodies. Western blot analysis of immunoprecipitated proteins revealed that PDI was co-precipitated by anti-SOD1 antibody and that SOD1 was co-precipitated by the anti-PDI antibody. This result suggested a physical interaction between PDI and SOD1. Fainter PDI bands were observed in the OGD/reperfusion group after immunoprecipitation using the anti-SOD1 antibody. The up-regulated PDI after OGD/reperfusion treatment seemed not to bind any more SOD1 in the immunoprecipitation studies. The reverse experiment was also performed: the cell lysates were immunoprecipitated with anti-PDI antibody, followed by Western blot with anti-SOD1 antibody. The SOD1 bands were observed, which were also more abundant in the control group (Figure 2C).

### PDI is S-nitrosylated in astrocytes following OGD/reperfusion; this S-nitrosylation of PDI is blocked by iNOS inhibitor 1400W

**Figure 3:**
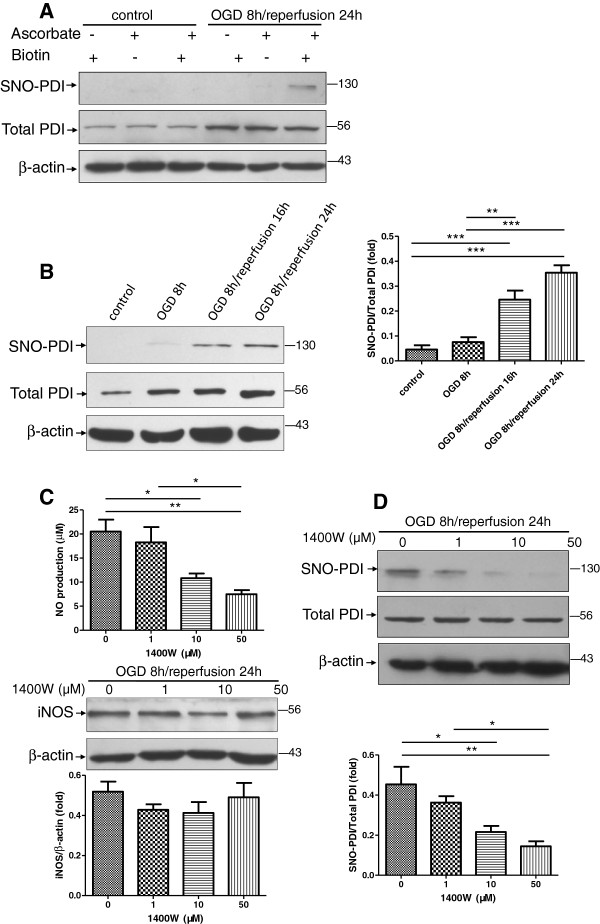
**S**-**nitrosylation of PDI in astrocytes following OGD**/**reperfusion.A**. In the presence of both ascorbate and biotin-HPDP, astrocytes following OGD 8 h/reperfusion 24 h treatment had detectable SNO-PDI, indicating the specificity of biotin-switch assay. **B**. PDI was S-nitrosylated in cultured astrocytes following OGD/reperfusion. Densitometric quantitation showed significant differences of SNO-PDI levels between the control group and the OGD/reperfusion groups. **C**. 1400W significantly inhibited NO production; the iNOS protein expression remained unaltered in cultured astrocytes following OGD 8 h/reperfusion 24 h. The cultured astrocytes were pretreated with various concentrations of iNOS inhibitor 1400W, which suppressed the S-nitrosylated PDI formation under OGD 8 h/reperfusion 24 h. The level of SNO-PDI was reduced with the increased concentration of 1400W (1, 10, and 50 μM). Moreover, 1400W suppressed S-nitrosylation of PDI, with the maximum effect seen at the concentration of 50 μM. Three independent experiments were performed. Data were presented as mean ± SEM; *, *P* < 0.05; **, *P* < 0.01; ***, *P* < 0.001 by one-way ANOVA (analysis of variance) with Tukey’s *post*-*hoc* test.

We investigated whether or not aberrant generation of NO through activation of iNOS mediated S-nitrosylation of PDI in reactive astrocytes following OGD/reperfusion. Using a biotin-switch assay, we identified that PDI was S-nitrosylated in cultured astrocytes after ischemia/reperfusion injury. The specificity of the biotinylation reaction was confirmed by no detection of SNO-PDI in the samples without the presence of ascorbate. Ascorbate is required to enhance the chemical decomposition of nitrosothiol groups required for reacting with the biotinylating reagent biotin-HPDP [28]. In addition, no detection of SNO-PDI in the absence of biotin-HPDP also confirmed the specificity of the final streptavidin precipitation step of the assay (Figure 3A). Despite the fact that total PDI levels were increased in astrocytes under OGD/reperfusion treatment, abundant SNO-PDI levels were detected in astrocytes following OGD 8 h/reperfusion 24 h treatment. However, SNO-PDI was virtually undetectable in the control group and the OGD 8 h group. This trend of SNO-PDI level was consistent with the change of iNOS expression and NO level during the OGD/reperfusion process (Figure 3B). To rule out the possibility that the detectable SNO-PDI in the OGD/reperfusion group resulted from the up-regulation of total PDI expression after OGD/reperfusion treatment, we deliberately increased total protein loading to enhance total PDI level in the control group. However, we could not detect the presence of SNO-PDI in the control group. Furthermore, in the OGD 8 h/reperfusion 24 h group, with manipulated less total PDI level owing to less total protein loading had detectable SNO-PDI. These results demonstrate that NO-mediated S-nitrosylation of PDI is a characteristic feature of astrocytes in response to ischemia/reperfusion injury. To determine whether iNOS plays a role in promoting the SNO-PDI formation, we pretreated primary astrocytes cultures with the iNOS inhibitor 1400W for 30 min followed by OGD/reperfusion. As expected, 1400W pretreatment strongly inhibited iNOS activity as demonstrated by dose-dependent suppression of NO production in reactive astrocytes. However, the iNOS protein levels were not affected by 1400W (Figure 3C). Immunoblot analysis of cell lysates revealed that NO-mediated SNO-PDI formation following OGD/reperfusion treatment in astrocytes was significantly blocked by iNOS inhibition, with the blockade behaving in a dose-dependent manner. These results suggest that iNOS signaling is involved in the SNO-PDI formation in astrocytes following OGD/reperfusion (Figure 3).

### OGD/reperfusion triggers formation of detergent/salt-insoluble ubiquitinated-protein aggregates, which is blocked by iNOS inhibitor 1400W

**Figure 4:**
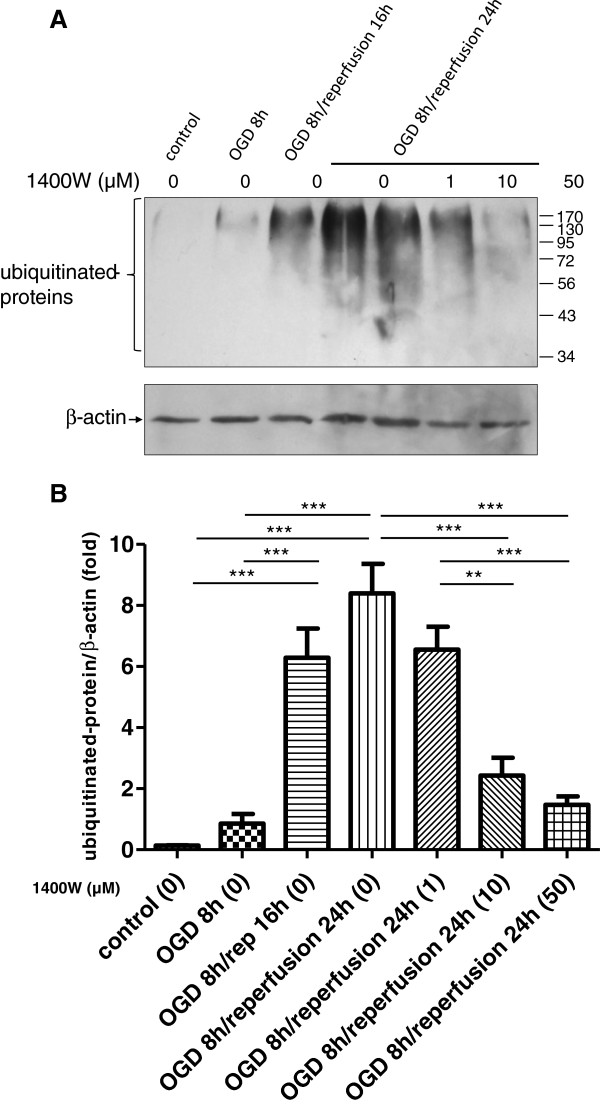
**Formation of detergent**/**salt**-**insoluble ubiquitinated**-**protein aggregates following OGD**/**reperfusion**, **and the inhibition of 1400W.A**. Protein smears ranging from 37 to 250 kDa were considered to be of ubiquitinated proteins. Increased levels of detergent/salt-insoluble ubiquitinated-protein aggregates were observed in pellet fractions under OGD/reperfusion. This change was more pronounced in OGD 8 h/reperfusion 24 h group. 1400W inhibited the formation of ubiquitinated-protein aggregates in the insoluble pellet fraction, with the maximum effect seen at the concentration of 50 μM. **B**. The ubiquitinated-protein aggregates were quantified using average intensity of protein smear, using β-actin as an internal control. Data from three independent experiments were presented as mean ± SEM; *, *P* < 0.05; **, *P* < 0.01; ***, *P* < 0.001 by one-way ANOVA (analysis of variance) with Tukey’s *post*-*hoc* test.

Protein aggregates have low solubility in the detergent/salt solution. We examined the formation of detergent/salt-insoluble ubiquitinated-protein aggregates in astrocytes under normoxic control conditions and after exposure to OGD/reperfusion. Under normal control conditions, the pellet fraction of astrocytes showed no or hardly any detectable ubiquitinated-protein aggregates. In contrast, during OGD/reperfusion treatment, there was a time-dependent accumulation of ubiquitinated proteins in the pellets. The ubiquitinated-protein smears ranged between 37 and 250 kDa, as detected by a monoclonal anti-ubiquitin antibody. Since these proteins were detergent/salt-insoluble, they were considered to be protein aggregates (Figure 4A). OGD led to a slight increase in ubiquitinated-protein aggregate formation. However, the difference between the OGD 8 h group and the control group did not reach statistical significance. The level of ubiquitinated-protein aggregates was further developed and reached approximately six-fold at 16 h reperfusion; it remained significantly elevated at 24 h reperfusion, at which point it was about eight-fold as compared with the OGD 8 h group (Figure 4B). These results suggest that OGD/reperfusion results in a progressive formation of ubiquitinated-protein aggregates. These aggregates may link to the formation of SNO-PDI in astrocytes. Since inhibiting the activity of iNOS through inhibitor 1400W led to the suppression of S-nitrosylation of PDI, we hypothesized that while S-nitrosylation of PDI was blocked by 1400W, the formation of ubiquitinated-protein aggregates might be subsequently inhibited. We examined the changes of ubiquitinated-protein aggregate levels in astrocytes following OGD 8 h/reperfusion 24 h treatment when S-nitrosylation of PDI was inhibited by 1400W. In the presence of various concentrations of 1400W, the levels of ubiquitinated-protein aggregates were significantly decreased in a dose-dependent manner. This change of ubiquitinated-protein aggregates with the use of 1400W correlated well with the change of SNO-PDI formation (Figure 4).

### OGD/reperfusion induces redistribution of ubiquitinated protein, and colocalization of ubiquitin with SOD1 protein

**Figure 5:**
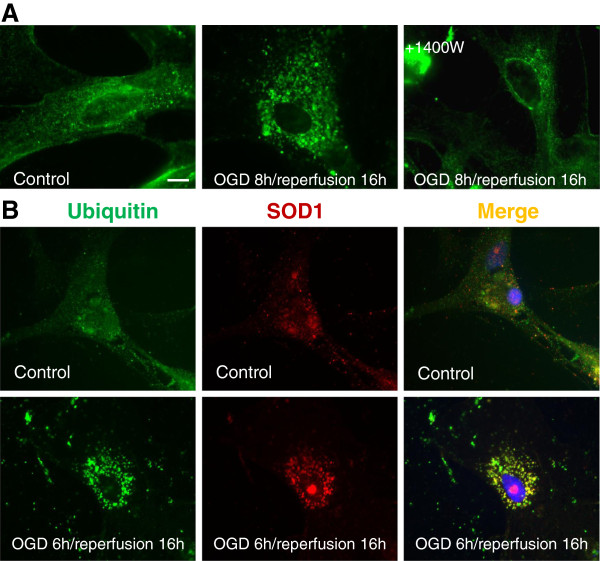
**Distribution of ubiquitin**-**conjugated proteins and SOD1 in cultured astrocytes following OGD**/**reperfusion.A**. In astrocytes, the ubiquitin immunoreactivity in the normoxic control group was evenly distributed in cytoplasm and nucleus. Following OGD 8 h/reperfusion 16 h, the diffused distribution of free ubiquitin lost nuclear staining and clustered immunoreactivity appeared near the nucleus. 1400W attenuated the punctuated ubiquitin perinuclear distribution **B**. Colocalization of ubiquitin and SOD1. Following OGD 8 h/reperfusion 16 h, were identified. SOD1 immunoreactivity colocalized with ubiquitinated-protein aggregates and showed a pattern of punctuated perinuclear-nuclear localization. Scale bar, 100 μm; green, ubiquitin immunosignals; red, SOD1 immunosignals; blue, nucleus staining with Hoechst 33342.

Since the monoclonal anti-ubiquitin antibody can recognize both free and conjugated ubiquitin, it was used to monitor both ubiquitin redistribution and formation. The normoxic control astrocytes showed even and diffuse immunoreactivity of ubiquitin with nuclear staining. In astrocytes subjected to OGD 8 h/reperfusion 16 h treatment, the diffuse distribution of free ubiquitin was absent; instead, the ubiquitin immunoreactivity changed into loss of nuclear staining and the appearance of aggregates throughout the cytoplasm. This punctuated ubiquitin in the perinuclear regions were considered to be the conjugated ubiquitin. With the use of 1400W to inhibit the S-nitrosylation of PDI, the punctuated staining of ubiquitin in the cytoplasm was less abundant when compared with those cells without 1400W treatment (Figure 5A). To investigate whether this ubiquitin was conjugated to SOD1 protein, we examined the localization of SOD1 under normal conditions and under conditions of OGD/reperfusion. Under normal conditions, SOD1 was distributed in the nucleus and throughout the cytosol. However, following OGD 8 h/reperfusion 16 h, the SOD1 immunoreactivity was clustered near nuclei in addition to the nuclear distribution. Small SOD1-positive aggregates were seen in the cytoplasm of astrocytes following OGD 8 h/reperfusion 16 h. To further examine the ubiquitination of SOD1, we performed double immunostaining with anti-SOD1 and anti-ubiquitin antibodies. After OGD 8 h/reperfusion 16 h treatment, the cultured astrocytes were immunostained with anti-SOD1 and anti-ubiquitin antibodies (Figure 5B). As a result, SOD1 aggregates induced by OGD 8 h/reperfusion 16 h were clearly colocalized with ubiquitin, indicating the ubiquitination of the SOD1 protein (Figure 5).

## Discussion

Brain ischemia/reperfusion injury encompasses all cell types in the central nervous system, including neurons and astrocytes. Astrocytes are believed to play a fundamental role in the pathogenesis of neuronal death. The failure of astrocytes in supporting the essential needs of neurons constitutes a great threat for neuronal survival. The multifaceted and complex role of astrocytes in response to injury includes the enhancement of neuronal survival or regeneration and contributes to further injury [29,30]. Glial cells, including astrocytes, generate excessive amounts of NO as a result of the activation of iNOS, and NO can induce neuronal apoptosis in ischemia/reperfusion injury. However, the cellular and molecular mechanisms of neuronal death induced by excessive NO have not yet been clearly defined. Brain hypoxic or ischemic injury is associated with an obvious inflammatory reaction that results in the expression and release of several cytokines [31]. These important mediators activate the expression of iNOS in different cell types, including glial cells in the central nervous system [32-34]. Interleukin-1β and tumor necrosis factor are both significantly increased within a few hours of ischemia. Interleukin-1β and tumor necrosis factor trigger transcriptional activation of the iNOS gene and then up-regulate iNOS expression [35,36]. Oxidative stress induced by ischemia might itself trigger the induction of iNOS. Moreover, the iNOS promoter contains a hypoxia response element, since a specific pathway, the hypoxia-inducible factor-1α pathway, can be activated at the onset of ischemia [37]. Consequently, the generation of NO persists. It is believed that NO produced by *de novo* expression of iNOS contributes to brain damage caused by hypoxic ischemia [38]. In the present study, we examined whether iNOS expression was enhanced in response to OGD/reperfusion in astrocytes. Consistently with previous research, OGD/reperfusion markedly elevated iNOS protein levels in cultured astrocytes. Our study gives the first demonstration that PDI is S-nitrosylated in cultured astrocytes following ischemia/reperfusion injury, and that this is highly associated with extensive generation of NO, which is induced by up-regulated iNOS expression. This finding suggests that S-nitrosylation of PDI probably inactivates the normal properties of PDI, and that it may contribute to the pathogenesis of ischemia/reperfusion injury.

Protein disulfide isomerase is a ubiquitous, highly conserved redox enzyme from the thioredoxin superfamily, located mainly in the ER [39]. During protein folding in the ER, PDI facilitates proper protein folding and helps to maintain the structural stability of the mature protein [17]. As a consequence, PDI is considered to be a molecular chaperone capable of stabilizing the correct folding of substrate proteins. It also facilitates the ER-associated degradation of misfolded proteins [40]. Protein disulfide isomerase is involved in the retro-translocation of misfolded cholera toxin from the ER to the cytoplasm by interacting with the ER transmembrane protein Derlin-1 [41]. In this study, we found that PDI expression was up-regulated in astrocytes following OGD/reperfusion. This result was consistent with previous studies that have demonstrated the up-regulation of PDI in astrocytes in response to hypoxia or transient forebrain ischemia in astrocytes [42]. A study of ischemic cardiomyopathy indicates that PDI is up-regulated in the viable peri-infarct myocardial region after infarction. This up-regulation of PDI led to a significant decrease in the rate of cardiomyocyte apoptosis [43]. All of this evidence put together indicates that the up-regulation of PDI in ischemia/reperfusion injury represents an adaptive response that promotes correct protein folding and offers potential protection to cells. However, detrimental generation of NO derived from iNOS induces S-nitrosylation of PDI; this posttranslational modification of PDI may attenuate its protective effects in ischemia/reperfusion injury.

As we know, ischemia-reperfusion causes accumulation of high-molecular weight ubiquitinated proteins following forebrain ischemia [44]. These ubiquitinated-protein aggregates are visualized in cultured astrocytes following glucose deprivation/recovery [14]. They are clustered with co-translational chaperones, protein folding enzymes [45], subcellular structures [46], proteasomes [47], and stress granules [48]. These changes may contribute to ischemic dysfunction of astrocytes and lead to neuronal damage. The accumulation of misfolded protein in the ER results in ER stress that triggers the protective unfolded protein response. The unfolded protein response entails the induction of chaperone molecules, the degradation of misfolded proteins, and the inhibition of protein translation [49]. Nonetheless, prolonged ER stress can still lead to activation of apoptosis [50]. Studies on pancreatic β cells, macrophages [51], and cerebellar granule cells [52] have demonstrated that NO can also induce ER stress. However, the molecular basis of this remains unknown. Furthermore, although the involvement of NO in the pathology of brain ischemia/reperfusion injury has been widely accepted, the chemical relationship between nitrosative stress and formation of ubiquitinated-protein aggregates has remained obscure. Our findings indicate that S-nitrosylation of PDI may hold some of the answers to these questions. Studies have shown that in Parkinson’s disease, excitotoxic activation of nNOS leads to excessive NO generation, which causes S-nitrosylation of the active-site thiols of PDI and inhibits its corresponding isomerase and chaperone activities [21]. In this way, NO blocks the protein’s protective effects via S-nitrosylation of PDI. S-nitrosylation of PDI leads to the accumulation of misfolded and polyubiquitinated proteins, and results in prolonged unfolded protein response activation. NO-mediated S-nitrosylation of PDI, therefore, participates in persistent ER stress and the induction of apoptosis [21].

We further demonstrated that NO-mediated S-nitrosylation of PDI may take part in the formation of ubiquitinated-protein aggregates in cultured astrocytes following OGD/reperfusion, since the aggregate’s formation was blocked by the iNOS inhibitor 1400W, which could efficiently inhibit the S-nitrosylation of PDI. When cultured astrocytes were subjected to OGD/reperfusion, the cells formed smear detergent/salt-insoluble ubiquitinated-protein aggregates. Furthermore, diffuse free ubiquitin staining changed into punctuated staining within perinuclear regions. This conjugated ubiquitin with reduced cytosolic and nuclear free ubiquitin distribution was considered to be an ubiquitinated-protein aggregate. The formation of these aggregates correlated well with the level of S-nitrosylation of PDI. With the use of 1400W to inhibit the activity of iNOS, the generation of NO was consequently decreased, which subsequently led to down-regulation of SNO-PDI levels. With the inhibition of S-nitrosylation of PDI, the formation of ubiquitinated-protein aggregates was decreased, since the detergent/salt-insoluble smear of ubiquitin in the pellet fraction was significantly reduced through the use of 1400W. This finding clearly demonstrates that blocking NO generation reduces the accumulation of ubiquitinated-protein aggregates. This blockade effect of 1400W may, result from reducing NO-mediated S-nitrosylation of PDI.

Free radicals contribute to neuronal death following hypoxic/ischemic brain injury. Not surprisingly, several studies have demonstrated that antioxidant treatment improves neuroprotection and recovery after brain injury [53-55]. SOD1 is an enzyme that detoxifies free radicals under normal physiologic conditions. SOD1 converts the superoxide anion into hydrogen peroxide, which is subsequently detoxified to water by glutathione peroxidase or catalase [56]. Reperfusion following cerebral ischemia leads to an overproduction of free radicals and the consumption of endogenous antioxidants. Neurons are particularly vulnerable to free radical damage, partly because of their relatively low levels of endogenous antioxidants. Studies have shown that non-neuronal cells may participate in free radical scavenging during ischemia/reperfusion [57]. One facet of reactive astrocytes in brain ischemia/reperfusion injury is the chronic secretion of antioxidants for neuronal protection and survival. SOD1 is one of the beneficial antioxidants produced by astrocytes. Prior studies using transgenic animal models have clearly established a beneficial role of SOD1 in adult ischemia/reperfusion injury [58]. Rodents overexpressing SOD1 have a much better outcome following head injury [59]. In our study, the expression of SOD1 was up-regulated in cultured astrocytes following OGD/reperfusion. The increased expression of SOD1 may represent a protective response to ischemic stress that enhances the antioxidant ability. However, studies have shown that SOD1 overexpression offers no protection under OGD conditions in a hippocampal culture model of excitotoxic injury [60]. Our results regarding the S-nitrosylation of PDI in cultured astrocytes following OGD/reperfusion provides an explanation to this finding. First, SOD1 was shown to be one of the PDI molecular targets [61] in ischemic cardiomyopathy. Second, a physical interaction between SOD1 and PDI has been indicated in cultured cells in familial amyotrophic lateral sclerosis [18]. Protein disulfide isomerase binds to both wild-type and mutant SOD1, and colocalizes with intracellular aggregates of mutant SOD1. Inhibition of the activity of PDI with the use of bacitracin increases aggregate production [18]. In patients with amyotrophic lateral sclerosis, PDI was found to be colocalized with SOD1 in neuronal cytoplasmic inclusions [62]. In this study, PDI and SOD1 were found to bind to one another in astrocyte cultures. Although PDI was up-regulated after OGD/reperfusion treatment, the increased total PDI did not bind more SOD1. Instead, less PDI-SOD1 binding was detected after OGD/reperfusion treatment in immunoprecipitation. It is possible that, despite the induction of PDI after ischemia/reperfusion injury, the SNO-PDI could not bind to SOD1 as efficiently as a normal PDI. In addition, SOD1 was ubiquitinated to form aggregates, and the insoluble SOD1 aggregates could not be detected in the soluble cell lysates used in the experiment. Some proportion of PDI was associated with SOD1 aggregates in the insoluble fraction of the cell lysates. We may suppose that PDI normally binds to SOD1 to form a disulfide-linked dimer. However, if PDI were S-nitrosylated, it could not bind to SOD1 as efficiently; and the disulfide-reduced SOD1 would more easily form aggregates. The diffuse distribution of SOD1 within the cytosol and nucleus under normal conditions changed into punctuated perinuclear and nuclear distribution following treatment with OGD 8 h/reperfusion 16 h. This result suggests abnormal folding of SOD1 in the cytoplasm had occurred. The ubiquitin-proteasome system (UPS) is the major intracellular proteolytic mechanism that controls the degradation of misfolded or abnormal proteins [63]. The colocalization of SOD1 and ubiquitin indicates that the misfolded SOD1 is ubiquitinated for further degradation.

## Conclusions

In this study, we have successfully demonstrated for the first time that OGD/reperfusion treatment in cultured astrocytes leads to an excess amount of NO generation by iNOS up-regulation in response to stress induced by ischemia/reperfusion. This leads to the formation of ubiquitinated-protein aggregates, probably through the process of S-nitrosylation of PDI. Our elucidation of an NO-mediated pathway that causes dysfunction of PDI by S-nitrosylation provides a mechanistic link between free radical production and abnormal protein aggregation in brain ischemia/reperfusion-induced injury. NO-based therapeutic strategies may help prevent aberrant protein misfolding by targeting the disruption or prevention of nitrosylation of specific proteins such as PDI. These therapeutic strategies may help improve the protective astrocytic activities in the future, thus enhancing neuronal survival, and improving the outcomes following brain ischemia/reperfusion injury.

## Abbreviations

ANOVA: Analysis of variance; BCA: Bicinchoninic acid; biotin-HPDP: *N*-(6-(biotinamido)hexyl)-3^′^-(2^′^-pyridyldithio)propionamide; EBSS: Earl’s balanced salt solution; NOS: Endothelial nitrogen oxide synthases; ER: Endoplasmic reticulum; iNOS: Inducible nitrogen oxide synthases; NO: Nitrogen oxide; nNOS: Neuronal nitrogen oxide synthases; NOS: Nitrogen oxide synthases; OGD: Oxygen glucose deprivation; PBS: Phosphate-buffered saline; PDI: Protein disulfide isomerase; SNO-PDI: S-nitrosylated PDI; SOD1: Copper-zinc superoxide dismutase.

## Competing interests

The authors declare that they have no competing interests.

## Authors’ contributions

XC, TG, and CL performed the experiments. XC, HS, LC, XML and JK conceived and designed the experimental plan and wrote the manuscript. All authors have read and approved the final version of the manuscript.
